# Investigating Cost Implications of Incorporating Level III At-Home Testing into a Polysomnography Based Sleep Medicine Program Using Administrative Data

**DOI:** 10.1155/2017/8939461

**Published:** 2017-07-16

**Authors:** Samuel Alan Stewart, Erika Penz, Mark Fenton, Robert Skomro

**Affiliations:** ^1^Medical Informatics, Department of Community Health and Epidemiology, Faculty of Medicine, Dalhousie University, Halifax, NS, Canada; ^2^Division of Respirology, Critical Care and Sleep Medicine, College of Medicine, University of Saskatchewan, Saskatoon, SK, Canada

## Abstract

**Objective:**

Obstructive sleep apnea is a common problem, requiring expensive in-lab polysomnography for proper diagnosis. Home monitoring can provide an alternative to in-lab testing for a subset of OSA patients. The objective of this project was to investigate the effect of incorporating home testing into an OSA program at a large, tertiary sleep disorders centre.

**Methods:**

The Sleep Disorders Centre in Saskatoon, Canada, has been incorporating at-home testing into their diagnostic pathways since 2006. Administrative data from 2007 to 2013 were extracted (10030 patients) and the flow of patients through the program was followed from diagnosis to treatment. Costs were estimated using 2014 pricing and were stratified by disease attributes and sensitivity analysis was applied.

**Results:**

The overall costs per patient were $627.40, with $419.20 for at-home testing and $746.20 for in-lab testing. The cost of home management would rise to $515 if all negative tests were required to be confirmed by an in-lab PSG.

**Discussion:**

Our review suggests that at-home testing can be cost-effective alternative to in-lab testing when applied to the correct population, specifically, those with a high pretest probability of obstructive sleep apnea and an absence of significant comorbidities.

## 1. Introduction

Obstructive sleep apnea (OSA) is a common chronic respiratory condition. In Canada, the diagnosed prevalence of the disease is 3% in adults, with an additional 19% at high risk [1]. Due to the growing obesity rates in Canada, OSA rates are on the rise [2] and if left untreated adverse effects can include increased cardiovascular morbidity and mortality, impaired cognitive function, and reduced quality of life [3–6].

The current gold-standard for OSA diagnosis is in-lab polysomnography (PSG), followed by treatment using continuous positive airway pressure (CPAP); however, at-home portable monitoring (PM) devices (level III testing) can provide an at-home alternative to in-lab testing and are an acceptable diagnostic method in a subset of OSA patients [7, 8]. There has been a significant growth in the use of PM devices for OSA diagnosis in Canada [9] but their cost effectiveness is unknown, given that home PM testing is less effective (inferior sensitivity and specificity) than PSG but potentially less costly [10].

Various health-economic assessments of OSA diagnostic pathways have been conducted [3–6, 10], but those focused on theoretical patient flows rather than real patient data. There are currently no data on the cost analysis of an OSA home management pathway in Canada.

The Sleep Disorders Center (SDC) for the Saskatoon Health Region in Saskatoon, Saskatchewan, Canada, has been using at-home testing in conjunction with in-lab testing for several years [9]. The purpose of this project is to perform a retrospective cost analysis evaluating the incorporation of at-home testing alongside in-lab PSG within a tertiary Canadian SDC, from the perspective of the healthcare payer. We have investigated the overall costs per patient and costs per CPAP prescription and have conducted sensitivity analyses to investigate the effects of potential changes to the existing patient flow.

## 2. Methods

We have previously published a paper on the effect of this workflow on patient wait-times [9] and ethics approval was obtained from the University of Saskatchewan Research Ethics Board (REB #13-135) for this research. The SDC at the Saskatoon City Hospital and the University of Saskatchewan developed a stream-lined process for triaging patients to either in-hospital testing (SL) or at-home testing and management (home monitoring, or HM) in 2006/07. Using standardized criteria, patients are assigned to either SL or HM after a visit with a Respirologist/sleep medicine physician. The criteria for referral to HM are as follows:

1. Absence of coexisting cardiac or respiratory disease.
2. Moderate to high pretest probability of OSA.
3. Low risk of hypoventilation.
4. Absence of coexisting sleep conditions such as insomnia or restless legs syndrome.
5. Ability to use level III technology at home with minimal supervision.

A more detailed description of the assignment process is available in our previous paper [9].

### 2.1. SL Pathway

In-lab polysomnography was performed as per standard criteria [11] using the Sandman version 9 in-lab PSG system (Mallinckrodt Inc., Canada). Split-night PSG with CPAP titration was typically performed in cases of moderate to severe OSA as per current AASM (American Association of Sleep Medicine) criteria [12]. Obstructive apnea was defined as decrease of flow by at least 90% for 10 seconds or more accompanied by respiratory effort, and hypopnea was defined as at least 30% decrease in flow of at least 10 sec duration accompanied by 3% desaturation or arousal [8]. OSA was defined as an AHI ≥ 5/hour as per the AASM definition [12].

### 2.2. HM Pathway

Cardiorespiratory home study was performed using an Embletta X10 (Embletta, USA) with the following channels: thermistor, pressure transducer, chest and abdominal effort channels, body position, heart rate, and oxygen saturation. A trained PSG technologist instructed the patient on the use of the device during a 30-minute teaching session. The patient was then provided with a device for use that night at home, to be returned the following morning. The study was scored by a PSG technologist: hypopnea was defined as a decrease in flow by 30% or more, lasting at least 10 sec with at least 3% desaturation; obstructive apnea was defined as a decrease of flow by at least 90% or more for at least 10 sec accompanied by respiratory effort [8]. All level III studies were reviewed by a sleep medicine physician for a clinical assessment and a decision was made whether to proceed with an Auto-CPAP titration (if there was clear evidence of OSA) or to suggest in-lab polysomnography to the referring physician. Level III studies were deemed acceptable if there was a minimum of 4 hours of data in all channels. OSA was defined as AHI ≥ 5/hour as per the AASM definition.

For those patients who proceeded with Auto-CPAP titration, a ResMed S8 or S9 (ResMed, USA) was used for a period of 7 days. Studies were of acceptable quality if the following criteria were met: Auto-CPAP unit was used at least 4 hours per night on at least 70% of the nights, average mask leaks were < 0.4 L/min, and residual apnea/hypopnea index downloaded from the Auto-CPAP device was less than 10 per hour. Patients that test negative at level III or who decide not to pursue Auto-CPAP titration are referred to SL for further testing if appropriate; otherwise they are referred back to their referring specialist.

Patients were reviewed by a sleep apnea Nurse Educator before and after Auto-CPAP titration. Auto-CPAP downloads were reviewed by a sleep medicine physician. If the Auto-CPAP titration was deemed acceptable and if the patient was willing to proceed with long term CPAP therapy, the patient was provided with a fixed-pressure CPAP unit at no charge (as per Sask. Provincial policy) at the P95 level of pressure derived from the Auto-CPAP titration on the same day as their posttitration appointment.

### 2.3. Costs of Diagnostic Pathways

Prices for the equipment and personnel involved with the HM and SL pathways are outlined in Table 1. At-home level III testing (including physician fee) costs $141 and titration with Auto-CPAP costs $197. PSG costs $384, $523, or $660 depending on whether the patient receives titration, diagnostics, or both during that visit (note that a patient's total cost will be either $660 for a split-night visit or $384 + $523 = $908 if the diagnostics and titration were performed on separate nights).

**Figure 1:**
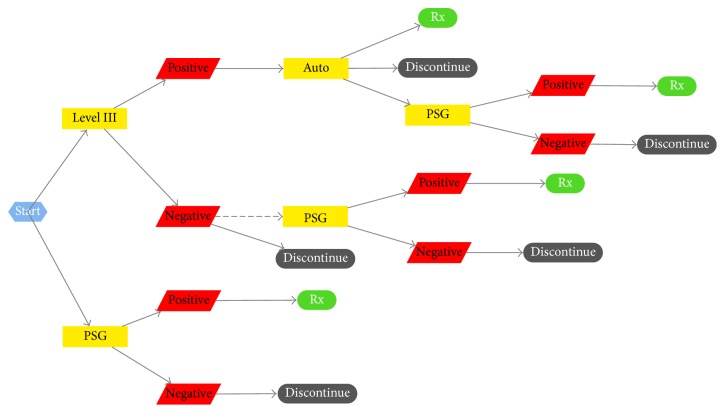
Theoretical patient flow within the sleep lab program.

**Figure 2:**
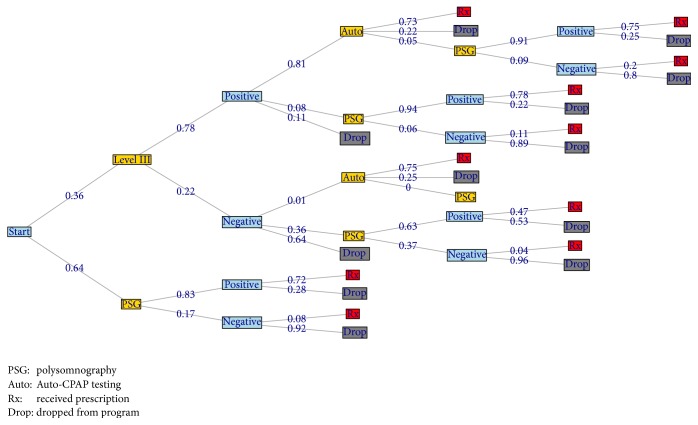
Patient flow through the SDC. The numbers on the lines in the figure indicate the proportion of patients that take that choice. This figure has been slightly simplified, as there is the potential for repeated procedures at most steps.

Theoretical flow of patients through the diagnostic pathways is presented in Figure 1 (the actual patient flows are presented in Figure 2). PSG patients are typically investigated with either two full nights PSG or 1 split night and then treatment is initiated with CPAP for positive tests, while HM patients undergo level III testing and, if positive, proceed to Auto-CPAP titration or, if negative, are referred to PSG lab for follow-up testing (at the discretion of the referring Respirologist, dotted line in Figure 1 indicates that pathway is not mandatory).

### 2.4. Data Extraction and Analysis

The SDC at the Saskatoon City Hospital maintains their own clinical database of all patients entering the program for either in-lab or at-home testing. Using this data, we were able to determine a patient's clinical pathway according to Figure 1.

Using the flow of patients, an average cost per patient, cost per CPAP prescription, and cost per discontinuation were calculated for each diagnostic pathway by multiplying the cost of testing performed in each pathway by the proportion of patients managed within the pathway. Overall costs per patient and per CPAP prescription were stratified by year of study and patient attributes (Apnoea-Hypnea Index (AHI), Epworth Sleepiness Score (ESS), patient BMI, and gender). The effects of patients discontinuing after level III testing were evaluated, and finally sensitivity to adjustments in patient flow was also explored.

## 3. Results

Table 2 presents the patient demographics for the sample population (all patients through the SDC in the Saskatoon Health Region from 2006 to 2013), and Figure 2 presents the flow of patients through the program. Note that Figure 2 is slightly simplified, as it does not show patients that had to repeat testing procedures (these are factored into the analysis). Level III was repeated 143 times (4%) normally due to data loss or short study duration (<4 hours). Auto tests were repeated more often (467 times, or 20%) usually due to poor adherence, poor adaptation to the machine, or high leak rates. PSG tests were repeated 665 times (9%), normally due to insufficient sleep (<4 hours) or suboptimal CPAP titration.

Table 3 presents the costs of both the HM and SL pathways, overall and stratified by disease and patient attributes. The table demonstrates that, overall, it costs approximately $327 less to diagnose a patient through HM compared to SL ($419 versus $746). Looking at costs to get patients to the end of the program, discontinued patients (i.e., patients that drop out of the program before a final diagnosis) are on average $100 cheaper ($464 versus $364) than patients that get CPAP prescriptions through HM, largely owing to those patients that discontinue after level III testing, while SL patients that discontinue tend to be $89 more expensive, since they are slightly more likely to receive full-night (i.e., 2 night) testing. While it is not possible to differentiate where SL patients drop, for HM patients the cost to treat patients that do not pursue treatment after their Auto is significantly higher than the cost to treat patients ($587 versus $465). This is due to the small percentage of post-Auto patients that are then tested in the SL, approximately 5% of Auto patients overall.

### 3.1. Sensitivity Analysis

#### 3.1.1. Adjusting HM Pathway Cost to Include Patients Who Discontinued Follow-Up within the HM Pathway

One challenge with the HM pathway is those patients that leave the program after a negative test: with the lower sensitivity of HM, there is a worry that more OSA may be going undetected; thus most patients that have a negative level III test are referred to PSG, but for a variety of reasons many do not pursue this second test. This is demonstrated in Figure 2, where 64% of patients drop from the HM after a negative test, and 11% drop after a positive test.  Table 4 compares the costs of patients in each pathway to the costs if all patients that did not go onto Auto-CPAP (i.e., dropout) completed in-lab testing instead. The HM costs increase from $420 to $516 if all negative tests were referred to and underwent a PSG and to $583 if all positive or negative level III tests that did not receive an Auto trial went to PSG. This demonstrates that, even if the most exhaustive diagnostic pathway was taken, the cost of patients through HM is still less than SL by $160.

#### 3.1.2. Probability of Testing Positive within HM Pathway

Another reason the HM pathway is less costly is the high positive level III test rate within the sleep lab, due to the HM pathway targeting patients with high pretest probability of OSA. Patients that test negative on level III often proceed to PSG afterward, so in this group of patients the level III test could be seen as an unnecessary test, at a cost of $141.  Table 5 investigates the costs to the program as the positive level III test rate drops from 75% to 20%. This approach assumes that all negative level III tests proceed to SL, similar to the second line in Table 4. The table demonstrates that every 5% drop in positive rate results in about a $20–$25 increase in costs per patient, but that the positivity rate would need to be very low (below 20%) for the cost of the HM pathway to exceed that of the SL pathway.

## 4. Discussion

Our review of the SDC patient flow and costs associated with HM and SL testing for patients suspected of obstructive sleep apnea suggests that incorporating a HM pathway into a SDC alongside a SL testing system can save money in the diagnosis of sleep apnea, compared with a system that diagnoses all diseases using PSG.

The HM pathway is successful largely because the physicians working within the SDC identify patients for HM with a high pretest probability for mild or moderate OSA. Providing a more affordable avenue for diagnosing and treating those patients with highly predictable and manageable disease allows the sleep lab to focus on more complex disease that cannot be tested and managed at home. This can be seen in the stratified costs in Table 3, where AHI, ESS, and BMI stratification all demonstrated that the more traditional OSA patients (moderate AHI and ESS values and overweight/obese patients) are the most affordable to send through HM.

It is important to consider the ramifications for false negative test results within a program such as this. In their 2011 paper, Pietzsch and colleagues [10] found the 10-year cost of untreated OSA was around $9,500 and the lifetime costs were almost $49,000. When considering such large costs associated with untreated OSA, the risk of a false negative is significant. To date, the literature has established that level III testing is less sensitive and less specific than in-lab testing [6, 13–15]. In the most extreme scenario, where every negative level III test is followed by an in-lab test to confirm a diagnosis of OSA, our analysis still demonstrates that the HM pathway has a cheaper per-patient cost than the SL pathway in a population with a moderate pretest probability of OSA.

HM is not appropriate for all patients, and the 75% positive level III test rate indicates that patients referred to the Sleep Well Program have at least moderate pretest probability of OSA. Our analysis demonstrated that the HM pathway would still be effective if that 75% rate dropped significantly, but at a cost to the program of an additional $20 per patient, or several thousand dollars annually.

This study* does not suggest that all patients should be managed with HM* or that HM is superior to PSG. The HM pathway is rigorously monitored within the SDC, with entry to it only allowed after consultation with a certified Respirologist. The HM pathway is designed for management of patients with high pretest probability of mild or moderate OSA, in an attempt to divert simple OSA cases away from in-lab testing. What this research does suggest is that diverting cases that have a high pretest probability of mild or moderate OSA to home testing instead of in-lab testing can be less costly and preserve valuable resources without significant negative effects on patient management.

Future research generated from this project is plentiful. There are pathways that are of interest in the flow of patients from Figure 1, including patients that follow that most expensive pathway mentioned above and investigating what mechanisms may be at play there that could be valuable in improving the prescreening process. The prescription rate after diagnosis is low, but while we know that the drops after testing are a combination of decision to forgo treatment and diagnoses of non-OSA disease, we do not know the exact disease or know what is driving the decisions to forgo treatment, a decision made by several patients. The management of disease does not end at prescription, so further research into adherence to treatment along with subsequent hospitalization rates and opinions on treatment should be explored, to determine if the HM pathway has any effect on the patients after CPAP prescription. Finally, there is potential for new and more accurate sensors to improve the process further, including using a questionnaire approach such as the Berlin Questionnaire [16], Sleep Apnea Clinical Score [17], or the Elbow Sign Results [18] to identify patients with a high pretest probability of OSA so that level III testing can be skipped entirely and patients can proceed directly to Auto-CPAP management. This kind of system would need to be closely monitored at the beginning to ensure accuracy of results but could reduce both costs and patient burden significantly. The effectiveness of these interventions would depend on the sensitivity and specificity of the tests, failure rates, target population, jurisdiction, and so forth, but the potential to further reduce the costs and burden of testing is worth investigating further.

## Figures and Tables

**Table 1:** Costs within the sleep lab program at the SHR. All prices are reported in 2014 Canadian Dollars. The variability of PSG costs is because some patients get their titration and diagnosis on two separate nights, while others get it done in one night (a “split-night” visit).

Section (total cost)	Component	Cost	Explanation
Level III testing *($141)*	Tech consult	$29.25	$39 hourly, 45-minute appointment
	Disposable equipment	$6.83	Respiratory effort belts $1.23/m & cannula $3.08
	Embletta kit cost	$2.61	$469 for parts used 180 times before replacement
	Tech scoring	$38.99	Billable code
	Resp. interpretation fee	$51.50	Billable code
	Admin costs	$12.08	$24.15 hourly, 0.5 hours per patient

Auto-CPAP *($197)*	Machine cost	$7.54	Machine costs $1750, used once a week for 5 years with a 3% degradation in price over time
	RN visit	$86.80	$57.87 hourly, 1.5-hour appointment
	Resp. interpretation fee	$102.20	Billable code

PSG *($384, $523, or $660)*	Equipment	$14.04	Belts and cannula from above, acetone ($1.39) and collodion ($0.89)
	Overnight tech	$229.67	$39/hour, two patients for 11.78 hours per night
	Daytime tech	$48.74	$39 hourly, 1.25-hour appointment
	RN	$28.93	$57.87 per hour, 0.5-hour appointment
	Resp. fee (titration)	$137.00	This is a billable code
	Resp. fee (diagnostic)	$276.00	This is a billable code

**Table 2:** Patient demographics (overall and by diagnostic pathway). Continuous variables are presented as mean (sd), with categorical variables presented as counts and proportions.

	Overall	Sleep lab (SL)	Home management (HM)
	Mean (sd)	Mean (sd)	Mean (sd)
*BMI*	34.08 (8.2)	34.47 (8.8)	33.41 (7)
*ESS*	10.28 (5.1)	9.92 (5.2)	10.9 (4.9)
*Age*	52.56 (13.9)	53.15 (14.3)	51.55 (13)
*AHI*	28.56 (29.8)	29.05 (32.8)	27.76 (23.9)

	Count	Count (%)	Count (%)

*Gender*			
Male	6492	4205 (66)	2287 (63)
Female	3523	2165 (34)	1358 (37)
*BMI*			
(0,25]	874	598 (10)	276 (8)
(25,30]	2216	1353 (23)	863 (26)
(30,35]	2571	1539 (26)	1032 (31)
(35,40]	1726	1081 (18)	645 (19)
40+	1845	1310 (22)	535 (16)
*AHI*			
(0,5]	1689	1303 (23)	386 (11)
(5,15]	2508	1521 (26)	987 (27)
(15,30]	2049	1054 (18)	995 (28)
30+	3130	1904 (33)	1226 (34)
*ESS*			
[0,6]	2505	1767 (29)	738 (21)
(6,10]	2468	1600 (26)	868 (25)
(10,15]	2929	1737 (29)	1192 (34)
15+	1628	951 (16)	677 (19)

**Table 3:** Costs of the HM and PSG arms of the program (in Canadian Dollars), overall and stratified by final disposition, AHI, ESS, gender, and BMI. Rx represents the costs for patients that get a prescription, and discontinued represents those patients that left the program before they received a diagnosis, because of either a negative test or a decision to not pursue treatment.

	Total (sd; *n*)	HM (sd; *n*)	SL (sd; *n*)
*Overall*	$627.4 (272.99, 10022)	$419.2 (269.46, 3642)	$746.2 (191.78, 6380)
*Rx*	$623.9 (202.47, 5725)	$464.7 (220.94, 1998)	$709.3 (126.21, 3727)
*Discontinued*			
All drops	$632 (345.23, 4297)	$364 (309.84, 1644)	$798 (247.96, 2653)
Drop after test		$147 (27.97, 833)	—
Drop after treatment		$586.8 (309.5, 811)	—
*AHI*			
(0, 5]	$615.6 (247.47, 1689)	$401.4 (327.28, 386)	$679.1 (173.48, 1303)
(5, 15]	$602.2 (273.26, 2506)	$392.2 (256.1, 985)	$738.2 (183.04, 1521)
(15, 30]	$594.9 (272.64, 2041)	$407 (215.44, 987)	$770.9 (190.96, 1054)
30+	$663.2 (293.39, 3127)	$459.6 (290.59, 1223)	$793.9 (208.44, 1904)
*ESS*			
[0, 6]	$653 (271.23, 2506)	$441.6 (308.87, 739)	$741.4 (194.82, 1767)
(6, 10]	$643.2 (262.19, 2412)	$433.7 (251.69, 812)	$749.5 (194.75, 1600)
(10, 15]	$612.9 (275.02, 2929)	$416.9 (264.71, 1192)	$747.3 (187.22, 1737)
15+	$619.5 (275.93, 1624)	$427.6 (260.55, 673)	$755.4 (193.58, 951)
*BMI*			
(0, 25]	$626 (269.56, 872)	$426.4 (288.24, 274)	$717.5 (203.34, 598)
(25, 30]	$608.2 (270.57, 2214)	$417.8 (274.06, 861)	$729.3 (185.16, 1353)
(30, 35]	$626 (267.96, 2536)	$436.1 (262.73, 997)	$749 (187.43, 1539)
(35, 40]	$638.6 (282.72, 1723)	$419.4 (256.62, 642)	$768.7 (207.04, 1081)
40+	$680.4 (268.13, 1845)	$465.4 (291.4, 535)	$768.2 (200.05, 1310)
*Gender*			
Male	$635.7 (272.33, 6487)	$421.3 (270.5, 2282)	$752.1 (190.29, 4205)
Female	$611.7 (273.19, 3520)	$416.3 (268.22, 1355)	$734.1 (193.54, 2165)

**Table 4:** Costs with adjusted patient flow.

	Total	HM	SL	HM Rx	HM drop	PSG Rx	PSG drop
*Original*	$627.60	$420.29	$746.20	$464.69	$363.99	$709.29	$798.04
Neg → PSG	$662.28	$515.59	$746.20	$487.54	$554.5	$709.34	$798.08
Neg* or *Pos → PSG	$686.66	$582.60	$746.20	$522.87	$692.03	$709.34	$798.08

**Table 5:** Investigating the effect of reducing the positive level III test rate on patient costs.

Positive level III rate	Total	Level 3	PSG
*0.75*	$664.69	$522.22	$746.20
*0.7*	$671.95	$542.17	$746.20
*0.65*	$679.21	$562.12	$746.20
*0.6*	$686.47	$582.08	$746.20
*0.55*	$693.73	$602.03	$746.20
*0.5*	$701	$621.98	$746.20
*0.45*	$708.26	$641.93	$746.20
*0.4*	$715.52	$661.89	$746.20
*0.35*	$722.78	$681.84	$746.20
*0.3*	$730.04	$701.79	$746.20
*0.25*	$737.30	$721.74	$746.20
*0.2*	$744.56	$741.70	$746.20
